# Differential Recall Bias, Intermediate Confounding, and Mediation Analysis in Life Course Epidemiology: An Analytic Framework with Empirical Example

**DOI:** 10.3389/fpsyg.2016.01828

**Published:** 2016-11-23

**Authors:** Mashhood A. Sheikh, Birgit Abelsen, Jan Abel Olsen

**Affiliations:** Health Services Research Unit, Department of Community Medicine, University of TromsøTromsø, Norway

**Keywords:** mood, memory, state of mind, recall bias, intermediate confounding, mediation, childhood socioeconomic status, child abuse

## Abstract

The mechanisms by which childhood socioeconomic status (CSES) affects adult mental health, general health, and well-being are not clear. Moreover, the analytical assumptions employed when assessing mediation in social and psychiatric epidemiology are rarely explained. The aim of this paper was to explain the intermediate confounding assumption, and to quantify differential recall bias in the association between CSES, childhood abuse, and mental health (SCL-10), general health (EQ-5D), and subjective well-being (SWLS). Furthermore, we assessed the mediating role of psychological and physical abuse in the association between CSES and mental health, general health, and well-being; and the influence of differential recall bias in the estimation of total effects, direct effects, and proportion of mediated effects. The assumptions employed when assessing mediation are explained with reference to a causal diagram. Poisson regression models (relative risk, RR and 99% CIs) were used to assess the association between CSES and psychological and physical abuse in childhood. Mediation analysis (difference method) was used to assess the indirect effect of CSES (through psychological and physical abuse in childhood) on mental health, general health, and well-being. Exposure (CSES) was measured at two time points. Mediation was assessed with both cross-sectional and longitudinal data. Psychological abuse and physical abuse mediated the association between CSES and adult mental health, general health, and well-being (6–16% among men and 7–14% among women, *p* < 0.001). The results suggest that up to 27% of the association between CSES and childhood abuse, 23% of the association between childhood abuse, and adult mental health, general health, and well-being, and 44% of the association between CSES and adult mental health, general health, and well-being is driven by differential recall bias. Assessing mediation with cross-sectional data (exposure, mediator, and outcome measured at the same time) showed that the total effects and direct effects were vastly overestimated (biased upwards). Consequently, the proportion of mediated effects were underestimated (biased downwards). If there is a true (unobserved) direct or indirect effect, and the direction of the differential recall bias is predictable, then the results of cross-sectional analyses should be discussed in light of that.

## Background and theoretical considerations

Several psychoanalytic and cognitive theorists have proposed that the risk of psychopathology in adulthood may have roots in adverse childhood experiences (Beck, 1976; Arieti and Bemporad, 1980; Brewin, 1989; Safran, 1990; Blatt and Homann, 1992). The “stress diathesis” model proposes that if genetic dispositions and stress from life experiences exceed a certain threshold, mental disorders are likely to develop (Brietzke et al., 2012). Parental history of psychological problems can be used as a crude proxy for genetic predisposition to mental health problems. The complex interplay between genetic dispositions and social factors in early childhood can shape one's susceptibility, and can contribute to the development of disorders. For instance, exposure to severe stress caused by low socioeconomic status (such as malnutrition, interaction with stressed-out parents, etc.) in early childhood can make an individual more susceptible, both biologically and psychologically, to subsequent stress (such as abuse). Furthermore, some studies have suggested that childhood abuse may not occur at random, as children with behavioral problems and poor social conformity may have a tendency to put themselves in situations with a high probability of stressful events (Plomin et al., 1990; Lyons et al., 1993).

The chains of risk model (Ben-Shlomo and Kuh, 2002; Lynch and Smith, 2005) suggests that childhood socioeconomic status (CSES) influences mental health, general health, and well-being in adulthood through its influence on psychological abuse and physical abuse in childhood. Previous studies have shown that children from low socioeconomic backgrounds are at a higher risk of experiencing psychological abuse and physical abuse in childhood (Dubowitz et al., 1987; Whipple and Webster-Stratton, 1991; Belsky, 1993; Fergusson and Lynskey, 1997; Garbarino, 1999; Hussey et al., 2006; Currie and Spatz Widom, 2010; Schilling and Christian, 2014; Romens et al., 2015). In turn, psychological abuse and physical abuse in childhood may have a long-term impact on mental health (anxiety and depression), general health (health-related quality of life), and subjective well-being (Sheikh et al., 2016a). In this way, CSES may influence mental health, general health, and well-being in adulthood, partly through psychological abuse and physical abuse in childhood. However, we found no previous studies on the mediating role of psychological abuse or physical abuse in the association between CSES and mental health, general health, and well-being in adulthood.

Other potential factors influencing childhood abuse are cultural (e.g., higher societal tolerance toward the abuse of boys), situational (e.g., temperament of children) or familial (e.g., lack of tolerance or hostility in parents). Some previous studies have focused on only one childhood adversity (Kessler et al., 1997; Fuller-Thomson et al., 2012), but children exposed to one type of adversity are likely to be exposed to other types of adversities in childhood (Sheikh et al., 2016a). Since different adversities may be correlated and co-occur in the same individuals, we aim to address the question of whether there is an independent and unique effect of CSES, psychological abuse, and physical abuse in childhood on mental health, general health, and well-being in adulthood, a question which has been rarely addressed in previous studies (Sheikh et al., 2016a).

Most studies on mental disorders have reported higher rates of traumatic experiences among women. Two potential mechanisms may explain these differences: either women are more likely to be abused, or they are more likely to report that they were abused (and vice versa). Studies based on self-reported information cannot distinguish between these mechanisms. However, another possibility is to assess the gender differences in self-reported information on other childhood adversities, such as socioeconomic status. Provided that the sample is representative, men and women should have the same likelihood of growing up in a household with low socioeconomic status. Therefore, a gender difference in self-reported information on CSES may indicate women are indeed more likely than men to report their childhood adversities (or vice versa). In the present study, we assess the gender differences in reporting CSES, psychological abuse, and physical abuse in childhood.

Furthermore, several studies have shown that psychological abuse is associated with a greater negative influence on adult health, as compared to physical abuse (Ney, 1987; Teicher et al., 2006; Dias et al., 2014). However, these studies based their conclusions on very small study samples. In the present study, we assess and compare the influence of psychological abuse and physical abuse on mental health, general health, and well-being, using a representative sample of 10,325 respondents.

Many studies on the long-term effects of childhood adversities have focused either on a single psychiatric disorder or on self-rated health, but few studies have looked at generic measures of health-related quality of life (Edwards et al., 2003; Sheikh et al., 2014, 2016a) and subjective well-being (Sheikh et al., 2014, 2016a). Previous studies have shown that the single-item self-rated health question is an unreliable measure of health (Crossley and Kennedy, 2002; Zajacova and Dowd, 2011), in contrast to disease-specific or symptom-specific measures of health (Sheikh et al., 2016b). Similarly, over 70% of the variation in the single-item life satisfaction (well-being) question is driven by the mood of the respondent at the time the questions is asked (Seligman, 2012). Only one previous study (Sheikh et al., 2016a) assessed the influence of psychological abuse and physical abuse in childhood on a validated generic descriptive system for health-related quality of life, such as the EuroQol five dimension scale (EQ-5D). However, only the unweighted health profile (EQ-5D_profile_) was used (i.e., the sum of the five dimensions). In the present study, we assess the influence of CSES, psychological abuse, and physical abuse in childhood on preference-based health-related quality of life in adulthood (EQ-5D_utility_).

## Role of intermediate confounding and differential recall bias in assessing mediation

### Intermediate confounding

**Figure 1:**
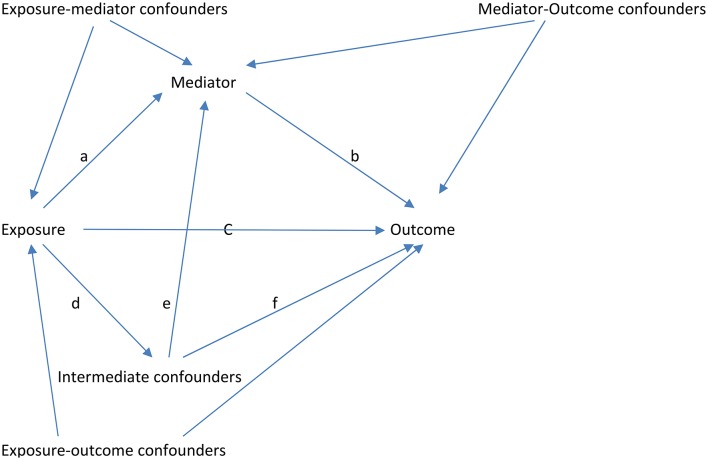
**Causal diagram of the hypothesized effects of exposure, mediator, confounders, and intermediate confounders on outcome**.

Most previous studies have assessed the mediating role of socioeconomic and behavioral factors in adulthood in the association between CSES and mental health, general health, and well-being in adulthood (Peck, 1994; Gilman, 2002; Galobardes et al., 2004, 2008; Pudrovska and Anikputa, 2012; Morgan et al., 2014; Sheikh et al., 2014). However, when assessing mediation, one assumes that there are no intermediate confounders (Cole and Hernán, 2002; Robins, 2003; Loeys et al., 2014; Sheikh et al., 2014; Tchetgen Tchetgen, 2014). Put simply, it is assumed that there are no variables (measured or unmeasured) that affect both the mediator(s) and the outcome, or that are affected by the exposure itself (see Figure 1). CSES may affect health in adolescence and early adulthood, which may in turn affect both socioeconomic status and health in adulthood. A wealthy upbringing and ample parental resources may expose children to experiences and styles of interacting that are useful for getting ahead in society. Similarly, behavioral factors in adulthood, such as alcohol and tobacco consumption are likely to be influenced by parental behavioral patterns and social environment in childhood (Sheikh et al., 2016a) and early adulthood. Due to the long time period between CSES, and socioeconomic status and behavioral factors in adulthood, there can be many potential intermediate confounders (Sheikh et al., 2014). Therefore, the “no intermediate confounding” assumption is difficult to satisfy. Such is the case when assessing mediators from adulthood in the association between CSES and health and well-being in adulthood.

Furthermore, the assumption of no “intermediate confounding” has rarely been explained graphically. The simplest (and broadest) definition of a confounder was given by Hernán (2008, p. 357) as “any variable that is necessary to eliminate the bias in the analysis” (see also Hernán et al., 2002). In the psychology literature, a confounder is referred to as the third (extraneous) variable (although, there is no limit on the number of potential confounders). Figure 1 presents the hypothesized association between the exposure (independent variable of interest), the mediator, potential confounders, intermediate confounders, and the outcome (dependent variable of interest). The intermediate confounder is a mediator-outcome confounder, as it influences both the mediator and the outcome (represented by paths *e* and *f*). Therefore under the “sequential ignorability” assumption (also referred to as “no unmeasured confounding,” “conditional exchangeability,” “selection on variables,” or “exogeneity” assumption), the intermediate confounder should be included in regression models as a confounder. However, the intermediate confounder is also influenced by the exposure (represented by path *d*) and acts as a mediator in the association between exposure and outcome (represented by path d^*^f). Therefore, if the intermediate confounder is included as a confounder to estimate the total effect (without controlling for the mediator), then the influence of exposure through the intermediate confounder is also blocked (path d^*^f). Similarly, the direct effect is supposed to represent the influence of an exposure on an outcome not mediated by the meditator. However, by adjusting for the intermediate confounder, part of the direct effect is blocked (path d^*^f), and hence the direct effect will be underestimated, as it represents the influence (represented by path *c*) of the exposure on the outcome not mediated by the mediator *and* the intermediate confounder. Moreover, the indirect effect (the difference between the total and direct effect) no longer represents the influence of the exposure on the outcome via the mediator, as the path d^*^e^*^b (note that the intermediate confounder acts as a *mediator* between exposure and mediator) is also blocked while estimating the direct effect. Therefore, the indirect effect will also be underestimated. Since the intermediate confounder is also an *intermediary* (synonymous with *mediator*) in the association between the exposure and the mediator (path d^*^e) and between the exposure and the outcome (path d^*^f), it creates problems when trying to break down the total effect. In the present study, we assess the role of psychological abuse and physical abuse as mediators in the association between CSES (exposure) and mental health, general health, and well-being outcomes in adulthood. Assuming that CSES is likely to *precede* psychological abuse and physical abuse in childhood, the relatively short time period between CSES and psychological and physical abuse in childhood allows us to avoid intermediate confounding.

### Differential recall bias

It is well established that *measurement error* (or *misclassification bias*) in the retrospective measurement of an exposure is a concern in observational studies. A distinction must be drawn between inaccurate recall (non-differential) and recall which is biased (differential). If the error in CSES is independent of the health and well-being outcomes, conditional on confounding variables, than it is considered non-differential, i.e., it may be systematic (concern for validity), but not with regard to the health/well-being status of respondents. Measurement error in the exposure (CSES) due to health/well-being in adulthood is considered “differential measurement error bias” (commonly referred to as *recall bias*), if the error in CSES depends on the subject's health/well-being at the time they participated, conditional on the confounding variables. As Mausner et al. (1985) noted, “*people may be more likely to search for explanations for the disease in the cases and, therefore, may assign more significance to past events.”* (Mausner et al., 1985, p. 165). On the other hand, *healthy* respondents may be less likely to recall a true exposure. Whether the *unhealthy* respondents recall their childhood socioeconomic conditions as worse than they were, or the *healthy* respondents recall their childhood socioeconomic status to be better, the aggregate effect of CSES on mental health, general health, and well-being will be biased upwards (overestimated). Furthermore, experts in mediation analysis propose that longitudinal data are suitable for the assessment of mechanisms or processes, as temporality is assumed between the exposure, the mediators, and the outcome (Hayes, 2013; Preacher, 2015). Much less attention is given to the question of how differential recall bias may bias estimates of total, direct, and indirect effects. No previous study has shown if there are any differences in estimates between analyses based on data from the same wave (cross-sectional data with exposure, mediator, and outcome measured at the same time point), and longitudinal data (exposure measured at an earlier time point). Self-reported information on childhood adversities, such as CSES may be influenced by the present mental health, general health, and well-being. For instance, if CSES is measured retrospectively at the same time point as health, *unhealthy* respondents may recall their CSES to be worse than it was (and vice versa), resulting in an overestimation of total and direct effects. However, no previous study has assessed the direction of differential recall bias for a *subjective* measure of childhood adversity, such as CSES and its influence on total, direct, and proportion of mediated effects. In this study we use the same exposure (CSES) measured at two time points, and compare the total, direct, and proportion of indirect effect estimates.

### Aims of the study

Using a representative sample from a large cohort study in Norway, we assessed: (i) the gender differences in self-reported CSES, psychological abuse, and physical abuse in childhood; (ii) the influence of CSES on psychological abuse and physical abuse in childhood; (iii) the influence of CSES, psychological abuse, and physical abuse on mental health (Hopkins Symptoms Check List-10, SCL-10), general health (EQ-5D), and subjective well-being (satisfaction with life scale, SWLS); (iv) the mediating role of psychological abuse and physical abuse in the association between CSES and mental health, general health, and well-being, and; (v) the role of differential recall bias in the estimation of total, direct, and proportion of mediated effects.

## Material and methods

### Study population

The Tromsø Study is a prospective cohort study which started in 1974 and contains data from 40,051 respondents. Tromsø Study participants are considered representative of the adult population residing in the municipality of Tromsø, Norway (Jacobsen et al., 2012). Between 1974 and 2007–2008, six waves of the Tromsø Study were conducted (referred to as Tromsø I–VI) (Jacobsen et al., 2012). The present study is based on 10,325 respondents participating in both Tromsø IV and Tromsø VI (aged 25–74 in Tromsø IV, and 38–87 in Tromsø VI).

### Measures of mental health, general health, and well-being

Mental health, general health, and well-being was measured in the Tromsø VI questionnaire. We transformed all outcomes on the same scale [0–10], so that meaningful comparison could be made between estimates.

Mental health status (anxiety and depression) was measured by the SCL-10, which is widely used in epidemiological studies. Respondents rated each of the 10 items in the SCL-10 on a four-point scale ranging from *not at all* (1) to *extremely* (4). We found an acceptable degree of internal consistency for the four-point scale in this sample (Cronbach's alpha 0.86, mean inter-item correlation: 0.41, McDonald's omega coefficient for composite reliability: 0.87). A composite variable was constructed as the sum of the 10 items. The total sum of scores were linearly transformed to lie on the scale [0–10], where 10 represented the worst mental health and 0 represented perfect mental health [mean: 0.89, standard deviation (SD): 1.20].

General health was assessed with the EQ-5D generic measure of health-related quality of life. Each health dimension has three levels: (1) no problems, (2) some problems, and (3) unable or extreme problems. Utility weights for EQ-5D_utility_ health states was based on the widely used UK value set (Dolan, 1997). These weights lie on a scale on which perfect health and being dead have a score of 1 and 0, respectively. The scores was linearly transformed to lie on the scale [0–10]; where 10 represented the lowest general health, and 0 represented perfect general health (mean: 1.36, SD: 1.61).

Well-being was measured by the response to the first three items on the SWLS (Diener et al., 1985; Sheikh et al., 2014, 2016a). These were “In most ways my life is close to my ideal,” “The conditions of my life are excellent,” and “I am satisfied with my life.” Respondents rated these statements using a 7-point scale ranging from *completely disagree* (1) to *completely agree* (7). A composite variable was constructed as the sum of the three items. The total sum of scores were linearly transformed to lie on the scale [0–10]; where 10 represented the lowest well-being, and 0 represented the highest well-being (mean: 2.68, SD: 1.98).

### Childhood socioeconomic status

Self-rated childhood financial condition was used as the indicator of CSES, and was measured in both the Tromsø IV and Tromsø VI questionnaires by the question, “How was your family's financial situation during childhood?” on a 4-point scale (1 = very good, 2 = good, 3 = difficult, 4 = very difficult). The test-retest reliability of CSES was good (Kappa_weighted_: 0.63, 99% CI: 0.61–0.65) in this sample (data not shown).

### Psychological abuse and physical abuse in childhood

Psychological abuse and physical abuse in childhood were used as mediators. Self-reported information on psychological abuse and physical abuse in childhood was collected in the Tromsø VI questionnaire by the question: “Have you over a long period experienced any of the following? (as a child).” The possible responses included: (i) “Being tormented, or threatened with violence” and; (ii) “Being beaten, kicked, or the victim of other types of violence.” Respondents could tick one or both responses. Those who ticked the first response were classified as having experienced psychological abuse in childhood, while those who ticked the second response were classified as having experienced physical abuse in childhood.

To assess whether there is an additive effect of childhood abuse on mental health, general health, and well-being, we constructed a separate variable of *Abuse frequency*: 0 = not exposed to psychological or physical abuse (reference), 1 = exposed to either psychological or physical abuse, 2 = exposed to both psychological and physical abuse.

### Confounders

Data on confounders were taken from the Tromsø IV questionnaire. The potential confounders age, gender, exposure to passive smoke in childhood (yes, no), living in Norway at age 1 (yes, no), mother's history of psychological problems, and father's history of psychological problems, were chosen based on *a priori* knowledge of the association between the exposures, mediators, and outcomes under study (Sheikh et al., 2014, 2016a). Mother's/father's history of psychological problems was measured as: “Does your mother/father have/has your mother/father ever had psychological problems?” (yes, no).

### Statistical analysis

All analyses were conducted using Stata version 13. Assuming that data was missing at random (MAR), imputed values were generated with multiple imputation (with chained equations), to avoid any bias in the association of interest introduced by excluding individuals with missing data. 100 multiple datasets are generated to help account for the uncertainty in the imputation procedure. In order to increase the predictive power of the imputation procedure, we included all indicators of mental health, general health, and well-being in the imputation models. A comparison between the complete-case (excluding missing) and the imputed dataset is presented with proportions (%), and mean (standard error, SE). All statistical analyses were performed on imputed dataset.

All confounding variables were tested for pairwise multiplicative interaction with CSES, psychological abuse, and physical abuse with logistic, Poisson, and linear regression (Ordinary least square, OLS) models. Statistically significant (*p* < 0.05) multiplicative interactions were observed with gender. Lower CSES among women was associated with a significantly lower mental health, general health, and well-being, as compared to men (data not shown). Similarly, psychologically or physically abused women had significantly lower mental health, general health, and well-being, as compared to men (data not shown). Therefore, associations between CSES, psychological abuse, physical abuse, mental health, general health, and well-being are presented separately for men and women. Robust standard errors were derived with bias-corrected accelerated bootstrapping (Carpenter and Bithell, 2000) and 99% confidence intervals (CIs) are reported. Both unadjusted (crude) and adjusted estimates are presented.

### Assessing gender difference in the exposure, mediators, and outcomes

We assessed whether there was a significant difference between men and women for the exposures, mediators, and outcomes under study (**Table 2**). Linear regression was used for continuous and ordinal variables (CSES, mental health, general health, and well-being), while Poisson regression (relative risk, RR) was used for childhood abuse (psychological abuse, physical abuse, and abuse frequency).

### Assessing the association between exposure and mediators, and mediators and outcomes

Poisson regression analysis (RR and 99% CIs) with robust error variance (Barros and Hirakata, 2003; Zou, 2004) was used to assess the association between CSES and psychological abuse, physical abuse, and abuse frequency in childhood (**Table 3**). To assess the linear association, *abuse frequency* was modeled as a continuous variable. In order to assess whether differential recall bias influences the association between CSES and psychological and physical abuse, the % *differences* between estimates of CSES in Tromso IV and Tromsø VI were also calculated, and 99% CIs are presented (**Table 3**). The differences between estimates of CSES in Tromsø IV and Tromsø VI were considered as a crude proxy for differential recall bias due to present mental health, general health, and well-being).

Linear regression models were used to assess the influence of psychological abuse, physical abuse, and *abuse frequency* in childhood on mental health, general health, and well-being in adulthood (**Table 4**). In order to assess whether differential recall bias in reporting psychological or physical abuse is present, the models were also adjusted for CSES in Tromsø VI. Five estimates are presented: unadjusted (model 1); adjusted for confounding variables and CSES in Tromsø IV (model 2); adjusted for confounding variables, CSES in Tromsø IV and psychological or physical abuse (model 3); adjusted for confounding variables, CSES in Tromsø IV, psychological or physical abuse, *and* CSES in Tromsø VI (model 4); and a difference between estimates from model 4 and 5 (% difference and 99% CIs) (**Table 4**).

To assess if there is an additive effect of psychological and physical abuse, we assessed the association between *abuse frequency* and mental health, general health, and well-being with linear regression models. In order to assess whether differential recall bias influenced the association between *abuse frequency*, and mental health, general health, and well-being, the *differences* between the estimates of abuse frequency adjusted for confounding varibles, CSES in Tromsø IV and adjusted for confounding variables, CSES in Tromsø IV and CSES in Tromsø VI were also calculated (**Table 5**). Four estimates are presented: unadjusted (model 1); adjusted for confounding variables and CSES in Tromsø IV (model 2); adjusted for confounding variables, CSES in Tromsø IV *and* CSES in Tromsø VI (model 3); and a difference between estimates from model 2 and 3 (% difference and 99% CIs) (**Table 5**).

### Assessing direct and indirect effects (through psychological abuse and physical abuse) of childhood socioeconomic status on mental health, general health, and well-being

The estimation strategy for assessing mediation was based on the chains of risk model and the Causal Steps method (Judd and Kenny, 1981; Baron and Kenny, 1986). We used the “difference method” approach (Wright, 1934; Judd and Kenny, 1981; Clogg et al., 1992) to assess mediation. An important assumption of assessing mediation is that there is no multiplicative exposure-mediator interaction (Clogg et al., 1992; Robins and Greenland, 1992; Have et al., 2004; Kaufman et al., 2004; Martinussen, 2009; Sheikh et al., 2014, 2016a). A multiplicative exposure-mediator interaction suggests that CSES interacts with psychological or physical abuse in its impact on mental health, general health, and well-being. Another assumption for assessing mediation with multiple mediators is that there is no multiplicative interaction between mediators (Lange et al., 2014; Sheikh et al., 2016a). No statistically significant (*p* > 0.1) multiplicative interaction between the exposure and mediators, or between the mediators was observed (data not shown). Psychological abuse and physical abuse were included in the models to assess the indirect effect and the proportion of mediated effect (%) (**Table 6**). Exposure (CSES) was measured at two time points (Tromsø IV and Tromsø VI). Mediation was assessed in both cross-sectional and longitudinal data. Four estimates are presented: total effects (adjusted for confounding variables), direct effects (adjusted for confounding variables and mediators), indirect effects, and proportion mediated (%) (**Table 6**). Indirect effect was calculated as:

(1) $$\begin{array}{r} \beta_{\text{Indirect effect}}=\beta_{\text{Total effect}}-\beta_{\text{Directeffect}} \end{array}$$

Proportion mediated (%) was calculated as:

(2) $$\begin{array}{r} \text{PM }\left(\text{\%}\right)\;=\;\frac{\beta_{\text{Total effect}}\;-\;\beta_{\text{Direct effect }}}{\beta_{\text{Direct effect}}\;+\;\beta_{\text{Indirect effect}}}*100 \end{array}$$

We calculated 99% CIs for all estimates using a bias-corrected accelerated bootstrap method (Carpenter and Bithell, 2000) with 500 re-samplings. Total effects, direct effects, indirect effects and proportion mediated were assessed with both CSES in Tromsø IV and CSES in Tromsø VI (**Table 6**).

Furthermore, the % difference in total effects and direct effects was calculated between estimates of CSES in Tromsø IV and CSES in Tromsø VI (**Table 7**). The % difference (H_0_: β_TE.T4_ = β_TE.T6_) in total effects was calculated as:

(3) $$\begin{array}{l} \%\;difference\;TE\;=\;\frac{\left(\beta_{\text{DE}.\text{T}6}\;+\;\beta_{\text{IE}.\text{T}6}\right)-\left(\beta_{\text{DE}.\text{T}4}\;+\;\beta_{\text{IE}.\text{T}4}\right)}{\beta_{\text{DE}.\text{T}6}\;+\;\beta_{\text{IE}.\text{T}6}}*100 \end{array}$$

Where β_DE_._T6_ is the direct effect of CSES (Tromsø VI), β_IE_._T6_ the indirect effect of CSES (Tromsø VI), β_DE_._T4_ the direct effect of CSES (Tromsø IV), and β_IE_._T4_ the indirect effect of CSES (Tromsø IV).

The % difference (H_0_: β_DE.T4_ = β_DE.T6_) in direct effects was calculated as:

(4) $$\begin{array}{l} \%\;difference\;DE\;=\;\frac{\left(\beta_{\text{TE}.\text{T}6}-\;\beta_{\text{IE}.\text{T}6}\right)-\left(\beta_{\text{TE}.\text{T}4}-\;\beta_{\text{IE}.\text{T}4}\right)}{\beta_{\text{TE}.\text{T}6}-\;\beta_{\text{IE}.\text{T}6}}*100 \end{array}$$

Where β_TE_._T6_ is the total effect of CSES (Tromsø VI), β_IE_._T6_ the indirect effect of CSES (Tromsø VI), β_TE_._T4_ the total effect of CSES (Tromsø IV), and β_IE_._T4_ the indirect effect of CSES (Tromsø IV).

Standard errors were derived with bias-corrected bootstrapping for hypothesis testing, and 99% CIs are presented.

## Results

Among the 10,325 respondents the majority were aged 45 years or older (61.9%) at Tromsø IV and 54% were women. More mothers than fathers were reported to have a history of psychological problems (6.5 and 2.5%, respectively). One-third (33.6%) of respondents reported having difficult or very difficult financial conditions in childhood (in Tromsø IV), while 7.9% reported psychological abuse, and 5.4% reported physical abuse in childhood (Table 1). However, the proportion of respondents reporting difficult or very difficult CSES was slightly lower (28.64%) in Tromsø VI.

**Table 1:** **General characteristics of the study sample (*n* = 10,325)**.

**Characteristics**	**Complete-case dataset *n* (%)**	**Imputed dataset (%)**
**AGE (IN 1994)**		
Mean (standard error, SE)	47.03 (0.12)	–^b^
25–34	1987 (19.2)	–^b^
35–44	1944 (18.8)	–^b^
45–54	3630 (35.2)	–^b^
55–64	2016 (19.5)	–^b^
65–74	748 (7.2)	–^b^
**GENDER**		
Men	4754 (46.0)	–^b^
Women	5571 (54.0)	–^b^
**HISTORY OF PSYCHOLOGICAL PROBLEMS, MOTHER**		
Yes	676 (6.5)	–^b^
No	9649 (93.5)	–^b^
**HISTORY OF PSYCHOLOGICAL PROBLEMS, FATHER**		
Yes	256 (2.5)	–^b^
No	10069 (97.5)	–^b^
**EXPOSURE TO PASSIVE SMOKE IN CHILDHOOD**^a^		
Yes	7589 (73.5)	73.5
No	2731 (26.5)	26.5
**LIVING IN NORWAY AT AGE 1**^a^		
Yes	8962 (97.9)	97.9
No	192 (2.1)	2.1
**CHILDHOOD SOCIOECONOMIC STATUS (TROMSØ IV)**^a^		
Mean (SE)	2.32 (0.01)	2.32 (0.01)
Very good	352 (3.7)	3.8
Good	5906 (62.6)	62.6
Difficult	2946 (31.2)	31.2
Very difficult	228 (2.4)	2.4
**CHILDHOOD SOCIOECONOMIC STATUS (TROMSØ VI)**^a^		
Mean (SE)	2.25 (0.01)	2.25 (0.01)
Very good	5.5	5.5
Good	65.9	65.7
Difficult	26.8	27.0
Very difficult	1.9	1.9
**PSYCHOLOGICAL ABUSE IN CHILDHOOD**		
Yes	818 (7.9)	–^b^
No	9507 (92.1)	–^b^
**PHYSICAL ABUSE IN CHILDHOOD**		
Yes	554 (5.4)	–^b^
No	9771 (94.6)	–^b^
**ABUSE FREQUENCY**		
Not exposed to psychological or physical abuse	9310 (90.2)	–^b^
Exposed to psychological or physical abuse	658 (6.4)	–^b^
Exposed to both psychological and physical abuse	357 (3.5)	–^b^
Mental health (SCL-10)^a^		
Mean (SE)	0.89 (0.01)	0.98 (0.01)
General health (EQ-5D)^a^		
Mean (SE)	1.36 (0.02)	1.43 (0.02)
Well-being (SWLS)^a^		
Mean (SE)	2.68 (0.02)	2.75 (0.02)

a *The numbers for some variables do not add up to 10,325 due to missing values*.

b *There were no missing values, so no imputations were made for these variables*.

*SCL-10, Hopkins Symptoms Check List-10; scale (0–10), where 0 represents perfect mental health, and 10 represents worst mental health*.

*EQ-5D, Euroqol-5Dimension generic measure of health-related quality of life; scale (0–10), where 0 represents perfect health, and 10 represents worst health*.

*SWLS, satisfaction with life scale; scale (0–10), where 0 represents highest well-being, and 10 represents lowest well-being*.

### Association between gender and childhood socioeconomic status, psychological abuse, physical abuse, mental health, general health, and well-being

Men reported a lower CSES (Tromsø IV) than women (β = −0.08, *p* < 0.001). The risk of psychological abuse in childhood among men was 20% (1/0.83 = 1.20) higher than that among women (*p* < 0.001), while the risk of physical abuse in childhood was 54% (1/0.65 = 1.54) higher among men than women (*p* < 0.001). The *abuse frequency* was linearly associated (*p* < 0.001) with lower mental health, general health, and well-being in adulthood. Women reported lower mental health (β = 0.29, *p* < 0.001) and general health (β = 0.28, *p* < 0.001), but higher well-being (β = −0.15, *p* < 0.001), as compared to men (Table 2).

**Table 2:** **Association between gender and childhood socioeconomic status (Tromsø IV), psychological abuse in childhood, physical abuse in childhood, mental health in adulthood, general health in adulthood, and subjective well-being in adulthood**.

	**Female (reference = male)**	
	**Unadjusted β (99% CI)**	**Adjusted β (99% CI)**
Childhood socioeconomic status (Tromsø IV)	−0.07 (−0.10, −0.04)^a^	−0.08 (−0.11, −0.05)^a^, ^b^
	**RR (99% CI)**	**RR (99% CI)**
Psychological abuse	0.76 (0.64–0.91)^a^	0.83 (0.71–0.97)^a^, ^c^
Physical abuse	0.59 (0.48–0.74)^a^	0.65 (0.53–0.79)^a^, ^d^
Abuse frequency	0.69 (0.58–0.81)^a^	0.60 (0.51–0.71)^a^, ^h^
	**β (99% CI)**	**β (99% CI)**
Mental health	0.46 (0.40–0.52)^a^	0.29 (0.24–0.35)^a^, ^e^
General health	0.52 (0.43–0.60)^a^	0.28 (0.21–0.36)^a^, ^f^
Subjective well-being	0.19 (0.08–0.30)^a^	–0.15 (–0.25, –0.04)^a^, ^g^

a *p < 0.001*.

b *Adjusted for age, parental history of psychological problems, exposure to passive smoke in childhood, living in Norway at age 1, psychological abuse, physical abuse, mental health, general health, and well-being*.

c *Adjusted for age, parental history of psychological problems, exposure to passive smoke in childhood, living in Norway at age 1, CSES in Tromsø IV, CSES in Tromsø VI, physical abuse, mental health, general health, and well-being*.

d *Adjusted for age, parental history of psychological problems, exposure to passive smoke in childhood, living in Norway at age 1, CSES in Tromsø IV, CSES in Tromsø VI, psychological abuse, mental health, general health, and well-being*.

e *Adjusted for age, parental history of psychological problems, exposure to passive smoke in childhood, living in Norway at age 1, CSES in Tromsø IV, CSES in Tromsø VI, psychological abuse, physical abuse, general health, and well-being*.

f *Adjusted for age, parental history of psychological problems, exposure to passive smoke in childhood, living in Norway at age 1, CSES in Tromsø IV, CSES in Tromsø VI, psychological abuse, physical abuse, mental health, and well-being*.

g *Adjusted for age, parental history of psychological problems, exposure to passive smoke in childhood, living in Norway at age 1, CSES in Tromsø IV, CSES in Tromsø VI, psychological abuse, physical abuse, mental health, general health, and well-being*.

h *Adjusted for age, parental history of psychological problems, exposure to passive smoke in childhood, living in Norway at age 1, CSES in Tromsø IV, CSES in Tromsø VI, mental health, general health, and well-being*.

### Influence of childhood socioeconomic status on psychological abuse and physical abuse in childhood

Table 3 presents the association between CSES in Tromsø IV, CSES in Tromsø VI, and psychological abuse, physical abuse, and abuse frequency. Lower CSES (Tromsø IV) was significantly (*p* < 0.001) associated with psychological abuse (RR = 1.85 among men and RR = 1.90 among women), physical abuse (RR = 1.60 among men and RR = 1.82 among women), and abuse frequency (RR = 1.74 among men and RR = 1.87 among women) in childhood (Table 3). The association between CSES and psychological abuse was stronger than that between CSES and physical abuse for both men and women (Table 3). Furthermore, the associations were stronger for women than men (Table 3).

**Table 3:** **The effect of childhood socioeconomic status on psychological abuse and physical abuse in childhood (*n* = 10,325)**.

	**Psychological abuse**		**Physical abuse**		**Abuse frequency**	
	**Model 1^a^**	**Model 2^b^**	**Model 1^a^**	**Model 2^b^**	**Model 1^a^**	**Model 2^b^**
	**RR (99% CI)**	**RR (99% CI)**	**RR (99% CI)**	**RR (99% CI)**	**RR (99% CI)**	**RR (99% CI)**
**MEN (*n* = 4754)**						
CSES (Tromsø IV)	1.55 (1.27–1.89)^c^	1.85 (1.51–2.25)^c^	1.45 (1.14–1.85)^c^	1.60 (1.25–2.05)^c^	1.51 (1.24–1.83)^c^	1.74 (1.43–2.11)^c^
CSES (Tromsø VI)	1.65 (1.36–2.00)^c^	1.88 (1.54–2.29)^c^	1.78 (1.42–2.22)^c^	1.91 (1.52–2.41)^c^	1.70 (1.42–2.04)^c^	1.89 (1.57–2.28)^c^
% difference (99% CI)^d^		2.65 (−7.67–16.78)		27.47 (5.95–39.49)^e^		13.53 (2.08–25.47)^e^
**WOMEN (*n* = 5571)**						
CSES (Tromsø IV)	1.66 (1.35–2.04)^c^	1.90 (1.53–2.36)^c^	1.61 (1.21–2.15)^c^	1.82 (1.35–2.46)^c^	1.64 (1.34–2.01)^c^	1.87 (1.51–2.32)^c^
CSES (Tromsø VI)	1.94 (1.60–2.36)^c^	2.12 (1.73–2.59)^c^	1.91 (1.45–2.50)^c^	2.07 (1.56–2.75)^c^	1.93 (1.59–2.34)^c^	2.10 (1.72–2.57)^c^
% difference (99% CI)^d^		14.45 (6.20–24.91)^e^		17.83 (5.25–32.29)^e^		15.73 (5.85–27.41)^c^

a *Mode 1, Unadjusted*.

b *Model 2, Adjusted for age, parental history of psychological problems, exposure to passive smoke in childhood, and living in Norway at age 1*.

c *p < 0.001*.

d *% difference between the estimate of CSES in Tromsø IV and Tromsø VI*.

e *p < 0.01*.

*Childhood socioeconomic status (CSES) was measured in Tromsø IV study (1994–1995), and Tromsø VI study (2007–2008), while psychological abuse and physical abuse was measured in Tromsø VI study (2007–2008)*.

*RR, relative risk*.

*CI, confidence interval*.

Confounding variables must not be a consequence of CSES, and lie on the causal path between CSES and psychological abuse and physical abuse in childhood. If confounding variables were influenced by CSES, the adjusted estimates would be weaker than the unadjusted estimates. The adjusted estimates were higher than the unadjusted estimates, indicating that none of the confounding variables act as mediator in the association between CSES and psychological abuse and physical abuse in childhood. This also suggests that confounding variables are not intermediate confounders in CSES→abuse→MH/GH/WB association. Generally, the association between CSES measured at the same time point (Tromsø VI) as the psychological or physical abuse, was stronger than the association with CSES measured at an earlier point in time (Tromsø IV) (Table 3). Among women, the % differences between estimates of CSES in Tromsø IV and Tromsø VI indicate that the association between CSES in Tromsø VI and psychological abuse was 14.45% (99% CI: 6.20–24.91) stronger. Similarly, the association between CSES in Tromsø VI and physical abuse was 27.47% (99% CI: 5.95–39.49) stronger among men, while it was 17.83% (99% CI: 5.25–32.29) stronger among women. Moreover, there was a pattern between men and women. The % differences indicate that psychologically abused women tended to report lower CSES in Tromsø VI than in Tromsø IV, while physically abused men tended to report lower CSES in Tromsø VI than in Tromsø IV (and vice versa). Furthermore, relative to the association between CSES in Tromsø IV and abuse frequency, the association between CSES in Tromsø VI and abuse frequency was 13.53% (99% CI: 2.08–25.47) stronger among men, while it was 15.73% (99% CI: 5.85–27.41) stronger among women (Table 3).

### Association between psychological abuse, physical abuse, and mental health, general health, and well-being in adulthood

Table 4 presents the association between psychological abuse, physical abuse, and mental health, general health, and well-being. Adjusted for confounding variables (Model 3), psychological abuse in childhood was associated with lower mental health (β = 0.38, *p* <0.001 among men *vs*. β = 0.54, *p* < 0.001 among women), general health (β = 0.18, *p* < 0.1 among men *vs*. β = 0.50, *p* < 0.001 among women), and well-being (β = 0.32, *p* < 0.01 among men *vs*. β = 0.67, *p* < 0.001 among women) in adulthood (Table 4). Similarly, physical abuse in childhood was associated with lower mental health (β = 0.21, *p* < 0.05 among men *vs*. β = 0.33, *p* < 0.01 among women), general health (β = 0.34, *p* < 0.01 among men *vs*. β = 0.37, *p* < 0.05 among women), and well-being (β = 0.21, *p* < 0.1 among men *vs*. β = 0.35, *p* < 0.05 among women) in adulthood (Model 3). Generally, psychological abuse in childhood had a greater influence than physical abuse on mental health and well-being in adulthood. However, among men, physical abuse in childhood led to lower general health in adulthood than psychological abuse (Model 3).

**Table 4:** **The effect of psychological abuse and physical abuse in childhood on mental health, general health, and well-being in adulthood (*n* = 10,325)**.

		**Model 1^a^**	**Model 2^b^**	**Model 3^c^**	**Model 4^d^**	**% difference in Model 3 and Model 4**
		**β (99% CI)**	**β (99% CI)**	**β (99% CI)**	**β (99% CI)**	**% (99% CI)**
**MENTAL HEALTH (SCL-10)**						
Psychological abuse	Men (*n* = 4754)	0.54 (0.36–0.72)^i^	0.48 (0.30–0.66)^i^	0.38 (0.18–0.59)^l^, ^i^	0.37 (0.08–0.54)^i^, ^j^	2.66 (0.89–11.85)^h^
	Women (*n* = 5571)	0.77 (0.53–1.00)^i^	0.69 (0.46–0.93)^i^	0.57 (0.32–0.82)^l^, ^i^	0.54 (0.44–0.66)^i^, ^j^	6.44 (4.98–8.65)^i^
Physical abuse	Men (*n* = 4754)	0.48 (0.29–0.67)^i^	0.43 (0.24–0.62)^i^	0.21 (−0.01–0.43)^e^, ^g^	0.19 (0.13–0.34)^h^, ^k^	11.29 (5.03–19.25)^h^
	Women (*n* = 5571)	0.73 (0.43–1.03)^i^	0.67 (0.37–0.97)^i^	0.33 (0.02–0.64)^e^, ^h^	0.31 (0.09–0.53)^h^, ^k^	6.17 (3.71–23.45)^h^
**GENERAL HEALTH (EQ-5D)**						
Psychological abuse	Men (*n* = 4754)	0.34 (0.12–0.57)^i^	0.33 (0.11–0.55)^i^	0.18 (−0.06–0.41)^l^, ^f^	0.16 (−0.00–0.41)^f^, ^j^	8.28 (2.74–88.72)^h^
	Women (*n* = 5571)	0.57 (0.30–0.84)^i^	0.64 (0.37–0.91)^i^	0.50 (0.21–0.80)^l^, ^i^	0.46 (0.28–0.66)^i^, ^j^	9.75 (6.90–11.99)^i^
Physical abuse	Men (*n* = 4754)	0.46 (0.19–0.73)^i^	0.44 (0.17–0.70)^i^	0.34 (0.05–0.62)^e^, ^h^	0.30 (0.14–0.40)^h^, ^k^	10.09 (6.33–24.77)^h^
	Women (*n* = 5571)	0.62 (0.26–0.98)^i^	0.67 (0.32–1.03)^i^	0.37 (−0.01–0.76)^e^, ^g^	0.34 (0.03–0.55)^h^, ^k^	7.28 (4.01–42.78)^h^
**WELL-BEING (SWLS)**						
Psychological abuse	Men (*n* = 4754)	0.51 (0.26–0.75)^i^	0.41 (0.16–0.66)^i^	0.32 (0.03–0.60)^l^, ^h^	0.30 (0.08–0.49)^h^, ^j^	6.59 (1.36–26.29)^h^
	Women (*n* = 5571)	0.87 (0.55–1.18)^i^	0.79 (0.48–1.11)^i^	0.67 (0.31–1.03)^l^, ^i^	0.61 (0.38–0.79)^i^, ^j^	8.74 (6.33–11.04)^i^, ^j^
Physical abuse	Men (*n* = 4754)	0.47 (0.19–0.74)^i^	0.39 (0.11–0.66)^i^	0.21 (−0.11–0.52)^e^, ^f^	0.16 (−0.02–0.32)^f^, ^k^	23.69 (11.76–203.89)^i^
	Women (*n* = 5571)	0.82 (0.43–1.21)^i^	0.74 (0.36–1.13)^i^	0.35 (−0.09–0.79)^e^, ^g^	0.32 (0.07–0.60)^h^, ^k^	9.19 (5.79–32.17)^h^

a *Model 1, Unadjusted*.

b *Model 2, Adjusted for age, parental history of psychological problems, exposure to passive smoke in childhood, living in Norway at age 1 and CSES in Tromsø IV*.

c *Model 3, Model 2+ adjusted for physical abuse when psychological was used as the exposure, and adjusted for psychological abuse when physical abuse was used as an exposure*.

d *Model 4, Model 3+ CSES in Tromsø VI*.

e *Adjusted for age, parental history of psychological problems, exposure to passive smoke in childhood, living in Norway at age 1, CSES in Tromsø IV + psychological abuse*.

f *p < 0.1*.

g *p < 0.05*.

h *p < 0.01*.

i *p < 0.001*.

j *Adjusted for age, parental history of psychological problems, exposure to passive smoke in childhood, living in Norway at age 1, CSES in Tromsø IV, physical abuse + CSES in Tromsø VI*.

k Adjusted for age, parental history of psychological problems, exposure to passive smoke in childhood, living in Norway at age 1, CSES in Tromsø IV, psychological abuse + CSES in Tromsø

l *Adjusted for age, parental history of psychological problems, exposure to passive smoke in childhood, living in Norway at age 1, CSES in Tromsø IV + physical abuse. VI*.

*SCL-10, Hopkins Symptoms Check List-10; scale (0–10), where 0 represents perfect mental health, and 10 represents worst mental health*.

*EQ-5D, Euroqol-5Dimensiongeneric measure of health-related quality of life; scale (0–10), where 0 represents perfect health, and 10 represents worst health*.

*SWLS, satisfaction with life scale; scale (0–10), where 0 represents highest well-being, and 10 represents lowest well-being*.

*RR, relative risk*.

*CI, confidence interval*.

In order to assess whether retrospective assessment of psychological abuse and physical abuse in childhood is influenced by recall bias, the estimates were further adjusted for CSES in Tromsø VI (Model 4), which was used as a crude proxy for differential recall bias (Table 4). The % differences in Model 3 and 4 indicate that some of the association between psychological abuse and mental health, general health, and well-being is driven by differential recall bias (2.66–8.28%, *p* < 0.05 among men *vs*. 6.44–9.75%, *p* < 0.001 among women). Similarly, after adjusting for CSES in Tromsø VI, the estimates for physical abuse in Model 3 were further attenuated (decreased) by 10.19–23.69%, *p* < 0.01 among men, and 6.17–9.19%, *p* < 0.01 among women. This indicates that even if CSES in Tromsø IV is included in the models, additional variables (such as CSES in Tromsø VI) should be included to account for differential recall bias (assuming that CSES reported in Tromsø IV does not influence reporting the CSES in Tromsø VI). Moreover, there was a pattern between men and women. The % differences indicate that women with lower mental health, general health, and well-being over-reported psychological abuse in childhood (and vice versa), while men with lower mental health, general health, and well-being over-reported physical abuse in childhood (and vice versa).

Table 5 presents the additive effect of psychological and physical abuse in childhood on mental health, general health, and well-being. Abuse frequency (Model 2) was associated (additively) with lower mental health and general health (*p* < 0.05). However, among men the association between psychological *or* physical abuse, and well-being was stronger (β = 0.44, *p* < 0.001) than with *both* psychological and physical abuse (β = 0.40, *p* < 0.001) (Model 2).

**Table 5:** **The effect of abuse frequency on mental health, general health, and well-being in adulthood (*n* = 10,325)**.

		**Model 1^a^**	**Model 2^b^**	**Model 3^c^**	**% difference in model 2 and 3**
		**β (99% CI)**	**β (99% CI)**	**β (99% CI)**	**% (99% CI)**
**MENTAL HEALTH (SCL-10)**					
Men (*n* = 4754)	**Abuse frequency**				
	Not exposed to psychological or physical abuse	Ref	Ref	Ref	Ref
	Exposed to psychological or physical abuse	0.42 (0.23–0.61)^e^	0.38 (0.19–0.57)^e^	0.36 (0.18–0.55)^e^	4.22 (2.47–5.92)^e^
	Exposed to both psychological and physical abuse	0.62 (0.37–0.87)^e^	0.55 (0.30–0.80)^e^	0.52 (0.27–0.76)^e^	5.73 (3.75–8.90)^e^
Women (*n* = 5571)	**Abuse frequency**				
	Not exposed to psychological or physical abuse	Ref	Ref	Ref	Ref
	Exposed to psychological or physical abuse	0.47 (0.24–0.71)^e^	0.41 (0.18–0.65)^e^	0.38 (0.15–0.61)^e^	7.93 (5.33–12.36)^e^
	Exposed to both psychological and physical abuse	1.08 (0.69–1.46)^f^	1.00 (0.61–1.39)^e^	0.94 (0.55–1.33)^e^	5.65 (0.99–10.30)^e^
**GENERAL HEALTH (EQ-5D)**					
Men (*n* = 4754)	**Abuse frequency**				
	Not exposed to psychological or physical abuse	Ref	Ref	Ref	Ref
	Exposed to psychological or physical abuse	0.18 (−0.04–0.41)^d^	0.19 (−0.31–0.41)^d^	0.17 (−0.05–0.38)^d^	12.24 (7.52–34.56)^e^
	Exposed to both psychological and physical abuse	0.58 (0.23–0.94)^e^	0.54 (0.20–0.89)^e^	0.50 (0.15–0.84)^e^	8.06 (5.47–17.29)^e^
Women (*n* = 5571)	**Abuse frequency**				
	Not exposed to psychological or physical abuse	Ref	Ref	Ref	Ref
	Exposed to psychological or physical abuse	0.45 (0.17–0.74)^e^	0.52 (0.24–0.80)^e^	0.48 (0.20–0.76)^e^	8.18 (5.25–16.78)^e^
	Exposed to both psychological and physical abuse	0.75 (0.30–1.21)^e^	0.83 (0.37–1.28)^e^	0.75 (0.29–1.20)^e^	9.60 (7.37–13.94)^e^
**WELL-BEING (SWLS)**					
Men (*n* = 4754)	**Abuse frequency**				
	Not exposed to psychological or physical abuse	Ref	Ref	Ref	Ref
	Exposed to psychological or physical abuse	0.50 (0.22–0.77)^e^	0.44 (0.17–0.72)^e^	0.41 (0.14–0.68)^e^	7.79 (4.21–11.86)^e^
	Exposed to both psychological and physical abuse	0.51 (0.17–0.86)^e^	0.40 (0.05–0.74)^e^	0.33 (−0.01–0.68)^e^	15.81 (10.38–57.49)^e^
Women (*n* = 5571)	**Abuse frequency**				
	Not exposed to psychological or physical abuse	Ref	Ref	Ref	Ref
	Exposed to psychological or physical abuse	0.63 (0.28–0.98)^e^	0.58 (0.23–0.93)^e^	0.53 (0.19–0.88)^e^	8.82 (0.87–16.77)^e^
	Exposed to both psychological and physical abuse	1.11 (0.64–1.58)^e^	1.02 (0.56–1.48)^e^	0.93 (0.46–1.39)^e^	8.66 (5.80–12.37)^e^

a *Model 1, Unadjusted*.

b *Model 2, Adjusted for age, parental history of psychological problems, exposure to passive smoke in childhood, living in Norway at age 1 and CSES in Tromsø IV*.

c *Model 3, Adjusted for age, parental history of psychological problems, exposure to passive smoke in childhood, living in Norway at age 1, CSES in Tromsø IV + CSES in Tromsø VI*.

d *p < 0.05*.

e *p < 0.001*.

*SCL-10, Hopkins Symptoms Check List-10; scale (0–10), where 0 represents perfect mental health, and 10 represents worst mental health*.

*EQ-5D, Euroqol-5Dimensiongeneric measure of health-related quality of life; scale (0–10), where 0 represents perfect health, and 10 represents worst health*.

*SWLS, satisfaction with life scale; scale (0–10), where 0 represents highest well-being, and 10 represents lowest well-being*.

*RR, relative risk*.

*CI, confidence interval*.

In order to assess whether *abuse frequency* is influenced by differential recall bias, the estimates were further adjusted for CSES in Tromsø VI (Model 3). The % differences between Models 2 and 3 indicate that some of the association between abuse frequency and mental health, general health, and well-being is driven by the differential recall bias (4.22–15.81%, *p* < 0.001 among men *vs*. 5.65–9.60%, *p* < 0.001 among women).

### Direct and indirect effect of childhood socioeconomic status on mental health, general health, and well-being in adulthood

Table 6 presents the total, direct and indirect effect of CSES measured in Tromsø IV and Tromsø VI. After adjusting for confounding variables, lower CSES (Tromsø IV) was significantly (*p* < 0.001) associated with lower mental health, general health, and well-being in adulthood for both men and women. Lower CSES (Tromsø IV) led to lower mental health and well-being among women, as compared to men. However, lower CSES (Tromsø IV) led to a greater negative impact on general health among men compared to women. Decomposition of total effects showed that both the direct effects and indirect effects were statistically significant (*p* < 0.001). Psychological abuse and physical abuse in childhood significantly mediated some of the association between CSES (Tromsø IV) and mental health, general health, and well-being in adulthood (6.46–16.43% among men and 7.38–14.07% among women, *p* < 0.001) (Table 6).

**Table 6:** **Direct and indirect effect (mediated through psychological abuse and physical abuse) of childhood socioeconomic status on mental health, general health, and well-being in adulthood**.

		**Total effect^a^**	**Direct effect^b^**	**Indirect effect^a^**	**Proportion mediated^a^**
		**β (99% CI)**	**β (99% CI)**	**β (99% CI)**	**% (99% CI)**
**MENTAL HEALTH (SCL-10)**					
CSES (Tromsø IV)	Men (*n* = 4754)	0.18 (0.13–0.19)^*^	0.15 (0.10–0.16)^*^	0.03 (0.01–0.04)^*^	16.43 (10.01–23.16)^*^
CSES (Tromsø VI)	Men (*n* = 4754)	0.26 (0.21–0.30)^*^	0.23 (0.17–0.27)^*^	0.03 (0.02–0.05)^*^	11.72 (8.38–15.46)^*^
CSES (Tromsø IV)	Women (*n* = 5571)	0.28 (0.26–0.36)^*^	0.25 (0.22–0.29)^*^	0.03 (0.03–0.04)^*^	12.12 (8.68–15.09)^*^
CSES (Tromsø VI)	Women (*n* = 5571)	0.38 (0.33–0.44)^*^	0.34 (0.30–0.41)^*^	0.04 (0.03–0.05)^*^	10.33 (7.47–12.32)^*^
**GENERAL HEALTH (EQ-5D)**					
CSES (Tromsø IV)	Men (*n* = 4754)	0.25 (0.22–0.30)^*^	0.23 (0.20–0.26)^*^	0.02 (0.01–0.03)^*^	8.62 (3.89–12.72)^*^
CSES (Tromsø VI)	Men (*n* = 4754)	0.36 (0.34–0.42)^*^	0.34 (0.31–0.39)^*^	0.02 (0.01–0.04)^*^	6.52 (3.31–9.17)^*^
CSES (Tromsø IV)	Women (*n* = 5571)	0.23 (0.18–0.28)^*^	0.20 (0.15–0.25)^*^	0.03 (0.02–0.04)^*^	14.07 (9.02–19.08)^*^
CSES (Tromsø VI)	Women (*n* = 5571)	0.41 (0.35–0.47)^*^	0.38 (0.32–0.40)^*^	0.04 (0.02–0.05)^*^	8.75 (5.98–10.77)^*^
**WELL-BEING (SWLS)**					
CSES (Tromsø IV)	Men (*n* = 4754)	0.38 (0.25–0.48)^*^	0.36 (0.23–0.46)^*^	0.02 (0.01–0.04)^*^	6.46 (4.34–9.76)^*^
CSES (Tromsø VI)	Men (*n* = 4754)	0.53 (0.40–0.64)^*^	0.50 (0.37–0.60)^*^	0.02 (0.01–0.03)^*^	4.73 (2.36–6.65)^*^
CSES (Tromsø IV)	Women (*n* = 5571)	0.53 (0.48–0.67)^*^	0.49 (0.44–0.62)^*^	0.04 (0.03–0.05)^*^	7.38 (5.85–10.33)^*^
CSES (Tromsø VI)	Women (*n* = 5571)	0.65 (0.58–0.74)^*^	0.61 (0.53–0.70)^*^	0.04 (0.04–0.07)^*^	6.82 (5.43–10.17)^*^

* *p < 0.001*.

a *Adjusted for age, parental history of psychological problems, exposure to passive smoke in childhood, and living in Norway at age 1*.

b *Adjusted for age, parental history of psychological problems, exposure to passive smoke in childhood, living in Norway at age 1, and psychological abuse and physical abuse in childhood*.

*SCL-10, Hopkins Symptoms Check List-10; scale (0–10), where 0 represents perfect mental health, and 10 represents worst mental health*.

*EQ-5D, Euroqol-5Dimension generic measure of health-related quality of life; scale (0–10), where 0 represents perfect health, and 10 represents worst health*.

*SWLS, satisfaction with life scale; scale (0–10), where 0 represents highest well-being, and 10 represents lowest well-being*.

*Childhood socioeconomic status was measured in Tromsø IV study (1994–1995), and Tromsø VI study (2007–2008), while mental health, general health, and well-being was measured in Tromsø VI study (2007–2008)*.

Assessing mediation through the use of CSES from the same wave (Tromsø VI) as the mediators and outcomes showed some differences. The direction of effects remained the same; however, the total and direct effects showed a stronger association, while the proportion of mediated (%) effects were weaker in magnitude (Table 6). This implies that assessing mediation with cross-sectional data biased the total and direct effects upwards, while the % mediated effects were biased downwards.

Table 7 presents the % difference in total effects and direct effects estimated with the CSES in Tromsø VI and the CSES in Tromsø IV (from Table 6). The total effect of CSES in Tromsø VI on mental health, general health, and well-being was stronger as compared to the total effect of CSES in Tromsø IV (27.89–32.14%, *p* < 0.001 among men *vs*. 18.99–44.42%, *p* < 0.001 among women). This indicates that differential recall bias has a substantial influence on the cross-sectional association between CSES and mental health, general health, and well-being. Moreover, the direct effect of CSES in Tromsø VI on mental health, general health, and well-being was stronger as compared to the direct effect of CSES in Tromsø IV (29.20–35.75%, *p* < 0.001 among men *vs*. 19.48–47.66%, *p* < 0.001 among women).

**Table 7:** **Role of differential recall bias in estimating total and direct effects**.

		**% difference in total effects^a^**	**% difference in direct effects^b^**
		**% (99% CI)^c^**	**% (99% CI)^c^**
	**MENTAL HEALTH (SCL-10)**		
	Men (*n* = 4754)	32.14 (23.38–43.66)^*^	35.75 (26.55–48.04)^*^
	Women (*n* = 5571)	26.85 (18.31–37.55)^*^	28.31 (19.37–40.52)^*^
	**GENERAL HEALTH (EQ-5D)**		
CSES	Men (*n* = 4754)	30.66 (25.95–40.75)^*^	32.21 (28.12–43.24)^*^
	Women (*n* = 5571)	44.42 (36.52–52.49)^*^	47.66 (38.50–57.30)^*^
	**WELL-BEING (SWLS)**		
	Men (*n* = 4754)	27.89 (21.94–41.28)^*^	29.20 (22.55–42.83)^*^
	Women (*n* = 5571)	18.99 (11.67–29.53)^*^	19.48 (11.20–31.02)^*^

* *p < 0.001*.

a *Adjusted for age, parental history of psychological problems, exposure to passive smoke in childhood, and living in Norway at age 1*.

b *Adjusted for age, parental history of psychological problems, exposure to passive smoke in childhood, living in Norway at age 1, and psychological abuse and physical abuse in childhood*.

c *The percentages show the proportion of difference in total and direct effects estimates using CSES in Tromsø IV and Tromsø VI as exposure*.

*SCL-10, Hopkins Symptoms Check List-10; scale (0–10), where 0 represents perfect mental health, and 10 represents worst mental health*.

*EQ-5D, Euroqol-5Dimension generic measure of health-related quality of life; scale (0–10), where 0 represents perfect health, and 10 represents worst health*.

*SWLS, satisfaction with life scale; scale (0–10), where 0 represents highest well-being, and 10 represents lowest well-being*.

*Childhood socioeconomic status was measured in Tromsø IV study (1994–1995), and Tromsø VI study (2007–2008), while mental health, general health, and well-being was measured in Tromsø VI study (2007–2008)*.

### Relative importance of CSES, psychological abuse, and physical abuse in childhood to mental health, general health, and well-being in adulthood

Statistically, the change in the estimate for exposure due to a confounder or a mediator may be assessed in the same manner (with either the “difference method” or statistical methods based on counterfactuals/potential outcomes). Therefore, the estimates for direct effects can be interpreted with an alternative hypothesis of whether CSES affects mental health, general health, and well-being in adulthood, independent of psychological abuse, and physical abuse in childhood.

**Figure 2:**
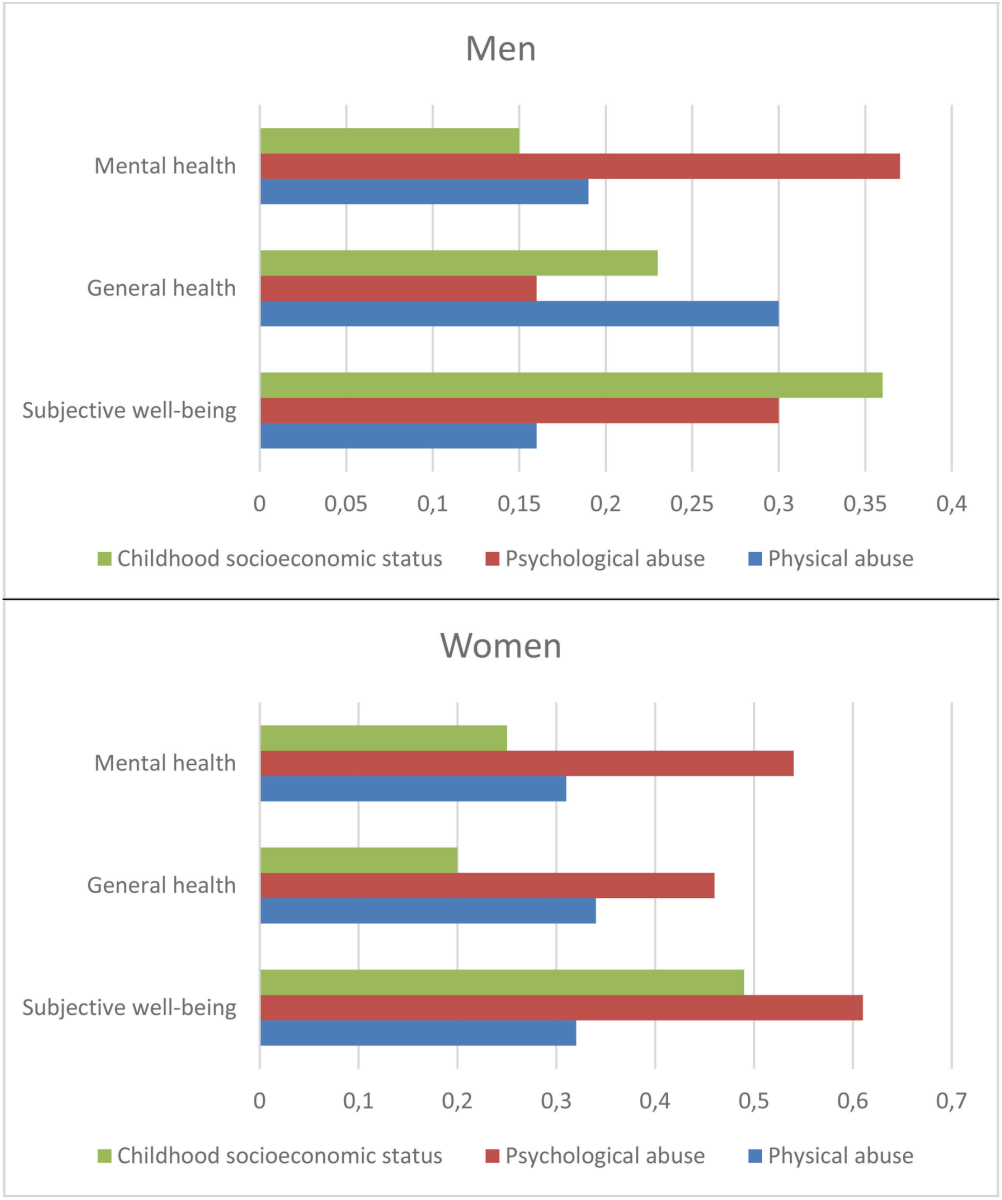
**Effect (OLS estimates) of childhood socioeconomic status (Tromsø IV), psychological abuse, and physical abuse in childhood on mental health, general health, and well-being in adulthood (adjusted for each other and confounding variables)**.

The estimates from model 4 in Table 4, and the direct effects from Table 6 (CSES from Tromsø IV), are presented in Figure 2 for comparison between the three childhood adversities. The relative impact of CSES, psychological abuse, and physical abuse in childhood to mental health was similar among men and women, i.e., psychological abuse had the strongest effect on mental health, followed by physical abuse, and CSES. However, for general health, physical abuse was most important among men, followed by CSES, and psychological abuse. For women, psychological abuse was most important, followed by physical abuse, and CSES. The pattern was different between men and women for well-being as well. For men, CSES had the strongest effect on well-being, followed by psychological abuse and physical abuse, while for women, psychological abuse was most important, followed by CSES and physical abuse (Figure 2).

## Discussion

This is the first study to assess the mediating role of psychological abuse and physical abuse in the association between CSES and mental health, general health, and well-being in adulthood. The results show that CSES has a direct as well as an indirect effect on mental health, general health, and well-being in adulthood. The indirect effects and proportion mediated imply that a substantial amount of the effect of CSES on adult mental health, general health, and well-being can be eliminated by interventions aimed at preventing psychological abuse and physical abuse in childhood. Although childhood abuse may occur to a different extent among boys and girls, even within the same family, the differences in *subjective* CSES reports indicate that men are more likely to report lower CSES than women (although the effect size was small) in this Norwegian sample. This might also suggest that contrary to previous studies, men are more likely to report psychological abuse and physical abuse in childhood.

Estimates of both CSES in Tromsø IV and Tromsø VI are presented for comparison purposes. The association between the exposure (CSES) and mediators measured at the same time point was stronger than the association with exposure measured at an earlier time point. This reflects that in a cross-sectional analysis, some of the association between CSES and psychological abuse and physical abuse in childhood is driven by recall bias (biased upwards).

The differential recall bias in this study indicates that among all the variables measured in Tromsø VI (CSES, psychological abuse, physical abuse, mental health, general health, and well-being), it may be that some of the associations are due to shared method variance, i.e., those with low mental health/general health/well-being are more likely to remember having a difficult childhood, both in terms of financial conditions and abuse suffered. Since psychological abuse and physical abuse were only measured in Tromsø VI, the differential recall bias in these variables could not be assessed comprehensively. However, the pattern of findings suggest that questions that aim to measure a *subjective* evaluation (such as CSES, which ranged from *very good* to *very difficult*) are strongly influenced by recall bias, while questions measuring concrete events (such as childhood abuse) might be less influenced by recall bias. For example, previous studies assessing the cross-sectional association of *subjective* CSES, fathers' education, and mothers' education (adjusted for one another) with adult health and well-being has shown that *subjective* CSES had the strongest influence (Mäkinen et al., 2006; Sheikh et al., 2014, 2016a). These findings might be driven by differential recall bias in *subjective* CSES, more so than the recall of parental education.

The results indicate that controlling for differential recall bias is important in observational studies. The main conclusion that can be drawn about the differences in estimates using CSES from two time points is that if there is a true (unobserved) direct or indirect effect, and the direction of the differential recall bias is predictable, than the results of cross-sectional analyses should be discussed in light of that. Using an empirical example, the results of this study indicate that research based on cross-sectional analyses between a *subjective* childhood adversity, and its influence on present mental health, general health, and well-being are vastly overestimated (biased upwards). Therefore, it is particularly important to note, that if the effect size is small, then the results (and consequently the inferences) are tentative. We recommend that future studies (using self-reported information) should adjust for a *subjective* evaluation of present or past attributes (such as *subjective* CSES) to account for recall bias.

Consistent with most previous studies (Dubowitz et al., 1987; Belsky, 1993; Fergusson and Lynskey, 1997; Garbarino, 1999; Hussey et al., 2006; Currie and Spatz Widom, 2010; Kendler and Aggen, 2014; Schilling and Christian, 2014; Romens et al., 2015), though not all (Connelly and Straus, 1992), lower CSES was associated with psychological or physical abuse in childhood. Moreover, consistent with previous findings, exposure to psychological abuse had a greater negative influence on mental health, and well-being, as compared to physical abuse in childhood (Dias et al., 2014; Sheikh et al., 2016a). Furthermore, the results of our study indicate that psychological abuse in childhood has a greater negative effect on mental health, general health, and well-being among women, as compared to CSES and physical abuse. However, among men, psychological abuse was most detrimental to mental health, physical abuse was most detrimental to general health, and CSES was most detrimental to well-being.

Similarly, consistent with previous findings (Schilling et al., 2007; Danese et al., 2009; Chartier et al., 2010; Carroll et al., 2013; Sheikh et al., 2016a), the three childhood adversities considered in this study (CSES, psychological abuse, and physical abuse) are independent predictors (adjusted for each other) of mental health, general health, and well-being in adulthood. This reflects that they cannot be used as proxies for one another (Sheikh et al., 2016a). More research is needed to understand how each of these indicators relate to other measures of physical and mental health in adulthood. These findings indicate the critical need for prevention and intervention strategies targeting adverse childhood experiences and their long-term mental health, general health, and well-being consequences.

An alternative explanation of the association between childhood abuse and adult mental health, general health, and well-being could be that the genetic risk factors for childhood abuse are associated with the genetic risk factors for lower mental health, general health, and well-being in adulthood. Previous studies on depression (Kendler et al., 1993; Kendler and Karkowski-Shuman, 1997) have shown this to be the case, however we did not find similar studies on health-related quality of life and subjective well-being.

The estimation of direct and indirect effects, and the causal interpretation require that there be no unmeasured exposure-outcome, mediator-outcome, or exposure-mediator confounders (Cole and Hernán, 2002; Robins, 2003). The standard regression approach to identify direct and indirect effects assumes sequential ignorability of the exposure and mediator(s), such that the exposure and mediators are effectively randomly assigned, given confounding variables (Imai et al., 2010). Some of the potential exposure-outcome and mediator-outcome confounders that are missing in the analysis are indicators of parental physical health, parental education, parental occupation, and neighborhood of residence. However, as Imai et al. noted (2011, p. 12), “*It is worth recalling that, in general, research with observational data is built upon a strong assumption that conditional on covariates the treatment variable is ignorable. Despite this, much can be learned from observational data. In fact, many social science theories result from simple observations and many experimental studies confirm the results of observational studies.”* (Imai et al., 2011, p. 12) Therefore, replication of this study's aim of assessing the impact of CSES on mental health, general health, and well-being, and the mediating role of childhood abuse is needed in other settings. Other assumptions for assessing mediation include “no interference,” implying that an individual's outcome is not influenced by the exposure status of another person (also referred to as SUTVA, the stable unit treatment value assumption).

The precise timing of CSES, psychological abuse, and physical abuse was not measured, and the temporality between these factors is assumed in this paper. The results of this study certainly do *not* imply that parents with low SES abuse their children, because information about the “abuser” was not measured, and psychological abuse and physical abuse were independent predictors of mental health, general health, and well-being in adulthood after controlling for CSES.

All outcomes were transformed into a [0–10] scale to facilitate comparison between the estimates in Figure 2. This by no means implies that a one-unit difference in subjective well-being is equivalent to a one-unit difference in mental health or health-related quality of life, but the effect size is comparable statistically, if not theoretically.

The strengths of this study are its large sample size, which is representative of the adult population of Tromsø. Furthermore, it includes three indicators of childhood adversities (CSES, psychological abuse, and physical abuse), and their association with three validated multi-item instruments of mental health, general health, and well-being. The methodological strength of the study is that due to the relatively small time-period between CSES, psychological abuse, and physical abuse in childhood, the intermediate confounding assumption (Cole and Hernán, 2002; Robins, 2003; Tchetgen Tchetgen, 2014) (also referred to as the “fourth assumption” or “no exposure-induced mediator-outcome confounder”) can be satisfied in this design.

In this study, the intermediate confounding assumption is explained with reference to social and psychiatric epidemiology. Using an empirical example from a large cohort study, the extent of differential recall bias in total, direct, and proportion of mediated effects is presented. The results show that total and direct effects were overestimated (biased upwards) in cross-sectional analysis. Consequently, the proportion of mediated effects were underestimated (biased downwards).

## What's known on this subject

Lower childhood socioeconomic status (CSES) is associated with lower mental health, general health, and well-being in adulthood. However, the mechanisms by which CSES affects adult mental health, general health, and well-being are not clear.

## What this study adds

Psychological abuse and physical abuse in childhood mediates the association between childhood socioeconomic status and mental health, general health, and well-being in adulthood. The findings suggest that associations between childhood adversity and adult mental health, general health, and well-being, based on cross-sectional analyses, are vastly overestimated (biased upwards). Therefore, controlling for differential recall bias is important in observational studies (using self-reported information).

## Ethical approval

The Tromsø Study has been approved by the Regional Committee for Medical and Health Research Ethics, the Data Inspectorate, and the Norwegian Directorate of Health. Written informed consent was obtained from all individual participants included in the study.

## Author contributions

MS conceived and designed the study, developed the theory, performed statistical analysis, data interpretation and wrote the manuscript. BA and JO contributed in the design of the project, and in the revision process of the manuscript.

## Funding

This research was funded by the University of Tromsø.

### Conflict of interest statement

The authors declare that the research was conducted in the absence of any commercial or financial relationships that could be construed as a potential conflict of interest.
